# Retinal Correlates of Cognitive Function in Early-Course Schizophrenia Spectrum Disorders: A Cross-Sectional Study

**DOI:** 10.1192/j.eurpsy.2025.415

**Published:** 2025-08-26

**Authors:** J. M. Gonzalez-Diaz, B. Sanchez Dalmau, A. Camos Carreras, S. Alba, S. Amoretti, F. Forte, M. Serra Navarro, S. Salmeron, A. Perez, C. Torrent, E. Vieta, M. Bernardo

**Affiliations:** 1 School of Medicine, Institute of Neurosciences – UBNeuro, Universitat de Barcelona, Barcelona, Spain; 2 CERSAME - UR Center for Mental Health, School of Medicine and Health Sciences, Universidad del Rosario, Bogota, Colombia; 3 Barcelona Clinic Schizophrenia Unit, Institute of Neurosciences, Hospital Clínic de Barcelona, Barcelona, Spain; 4 Clinica Nuestra Señora de la Paz – OHSJD, Bogota, Colombia; 5 Department of Ophthalmology, Hospital Clínic de Barcelona; 6 School of Medicine, Universitat de Barcelona; 7 Institut d’Investigacions Mèdiques August Pi i Sunyer (IDIBAPS); 8 Department of Neurology, Hospital Clínic de Barcelona, Barcelona; 9 Biomedical Research Networking Centre Consortium on Mental Health (CIBERSAM), Instituto de Salud Carlos III, Madrid; 10 Bipolar and Depressive Disorders Unit, Institute of Neurosciences; 11 Department of Psychiatry and Psychology, Institute of Neuroscience, Hospital Clínic de Barcelona, Barcelona, Spain

## Abstract

**Introduction:**

Emerging research suggests that retinal structure, assessed via optical coherence tomography (OCT), may be a potential biomarker in Schizophrenia Spectrum Disorders (SSD). However, the relationship between retinal and cognitive parameters in the early stages of the disease remains underexplored.

**Objectives:**

To examine the correlation between retinal structure and cognitive functioning in patients with early-course SSD.

**Methods:**

A cross-sectional sample of 26 SSD cases and 25 age- and gender-matched healthy controls (HCs) underwent OCT imaging. Peripapillary retinal nerve fiber layer (pRNFL), macular, and ganglion cell-inner plexiform layer (GCL+IPL) thicknesses were measured. Cognitive domains, including verbal memory (California Verbal Learning Test, CVLT[Delis et al. 2000]), working memory (WAIS-III Letter–Number Sequencing Subtest[Wechsler 2011]), processing speed (Trail Making Test-A, TMT-A[Reitan et al.Clin Neuropsychol 1995;9:50-6]), sustained attention (Conners’ Continuous Performance Test, CPT[Rosvold et al.J Consult Psychol 1956;20:343]), executive function (Wisconsin Card Sorting Test, WCST[Heaton 2008]), and social cognition (Mayer-Salovey-Caruso Emotional Intelligence Test, MSCEIT[Mayer et al.Emotion 2003;3:97-105]), were assessed and then transformed into t-scores. A Principal Component Analysis(PCA) was conducted, and associations between retinal and cognitive parameters were explored with Pearson/Spearman correlations; statistical significance was set at p<0.05.

**Results:**

SSD patients (mean age:31.9years[SD=1.2]; males n=11[44%]; mean duration of illness:32.5months[SD=22.3]) exhibited thicker pRNFL in both the right (t=-2.25,p=0.03) and left (t=-2.08,p=0.04) eyes compared to HCs (mean age:32.7years[SD=1.9]; males n=13[50%]). A thicker pRNFL was associated with a poorer cognitive performance: verbal (r=-0.53,p=0.04) and working memory (r=-0.64,p=0.01) was correlated with average pRNFL thickness; processing speed was associated with superior temporal pRNFL thickness (rs=-0.54,p=0.02); sustained attention was correlated with inferior nasal pRNFL thickness (rs=-0.54,p=0.04); social cognition was correlated with average pRNFL thickness (r=-0.72, p=0.03) and temporal pRNFL thickness (r=-0.82, p=0.01). Executive function was not associated with retinal measures, and macular and GCL+IPL thickness did not correlate significantly with cognitive variables.

**Conclusions:**

Our findings suggest relationships between increased pRNFL thickness and impaired cognitive functioning in early-course SSD patients. Previous studies have reported that pRNFL might be thicker during the initial stages of SSD and thins as the disease progresses, highlighting the role of inflammatory processes in both retinal changes and cognitive impairment. Further longitudinal multimodal research is warranted to explore the utility of retinal imaging in monitoring cognitive outcomes in SSD.

**Disclosure of Interest:**

None Declared

